# Three‐Dimensional Mapping of Distal Clavicle Fractures: Displacement Patterns and Clinical Implications for Surgical Management

**DOI:** 10.1111/os.70033

**Published:** 2025-03-24

**Authors:** Jinquan Liu, Jingyi Mi, Yesheng Jin, Fang Lin, Yongwei Wu, Yunhong Ma, Jun Liu, Zhonghua Xu, Li Tang, Aiping Zhu, Danfeng Jing, Yongjun Rui, Ming Zhou

**Affiliations:** ^1^ Department of Orthopaedics Wuxi Ninth People's Hospital Affiliated to Soochow University Wuxi China; ^2^ Department of Orthopaedics Changzhou Jintan First People's Hospital Changzhou China; ^3^ Suzhou Medical College of Soochow University Suzhou China

**Keywords:** computed tomography, distal clavicle fracture, fracture line, fracture mapping, Heatmap, three‐dimensional imaging

## Abstract

**Objective:**

Current classifications inadequately address distal clavicle fracture instability due to their coronal plane focus, neglecting multiplanar displacement and underestimation of complexity on routine radiographs. This study aimed to bridge this gap by employing three‐dimensional (3D) fracture mapping to characterize injury patterns, offering mechanistic insights to optimize surgical strategies.

**Methods:**

A retrospective analysis was conducted on 81 patients diagnosed with acute distal clavicle fractures at Wuxi Ninth People's Hospital between 2019 and 2022. Axial and sagittal CT planes were utilized to demonstrate fracture line alignment. Manual simulated repositioning was performed for all fracture lines, which were subsequently graphically superimposed onto a standard template of the intact distal clavicle. A 3D map was generated and subsequently transformed into a heatmap. The classification of distal clavicle fractures was determined based on the updated and modified Neer classification. Two points were designated at the distal end of the fracture block and at the repositioned counterpart to assess the three‐dimensional spatial position, including shortening along the x‐axis, horizontal displacement along the y‐axis, vertical displacement along the z‐axis, as well as the displacement angles in the three planes, thereby quantifying the displacement of each distal clavicle fracture.

**Results:**

This study included 81 cases of distal clavicle fractures (43 cases on the left side and 38 cases on the right side). The distribution included 8 cases (9.88%) of Neer I, 5 cases (6.17%) of Neer IIA, 31 cases (38.27%) of Neer IIB, 11 cases (13.58%) of Neer IIC, 14 cases (17.28%) of Neer III, and 12 cases (14.81%) of Neer V. Fracture mapping revealed that the fracture lines were predominantly located in the distal one‐third of the distal clavicle, with the highest concentration at the acromion end. The majority of displaced distal clavicle fractures exhibit multidirectional displacement, mainly posterior, superior, and shortening, along with angulation in the corresponding directions.

**Conclusions:**

Most displaced distal clavicle fractures involve multiple displacements and angulations, necessitating three‐dimensional analysis during fracture reduction. A comprehensive 3D assessment of displacement patterns is essential for evaluating stability and guiding treatment. Fracture line analysis further enhances classification accuracy and informs imaging protocols and fixation strategies tailored to specific fracture types.

## Graphical abstract

By establishing a fracture map, the fracture line distribution and fracture situation of distal clavicle fractures were evaluated, and the fracture types were further understood, which provided a basis for the preoperative application of imaging diagnosis methods and further development of surgical approaches and fixation technologies for specific fracture types.

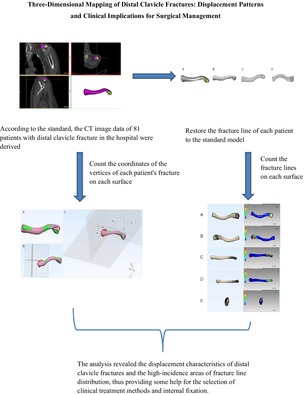

## 1 Introduction

Approximately 2%–5% of all fractures in adults involve the clavicle, with distal clavicle fractures constituting 10%–30% of these cases [1, 2, 3, 4]. Although less common than diaphyseal fractures, distal clavicle fractures pose a diagnostic and therapeutic challenge for shoulder surgeons. Nonoperative treatment of unstable distal clavicle fractures often leads to delayed healing or nonunion, exacerbated by the inherent difficulty in identifying the fracture pattern of occult unstable fractures [1, 2, 5, 6].

**FIGURE 1:**
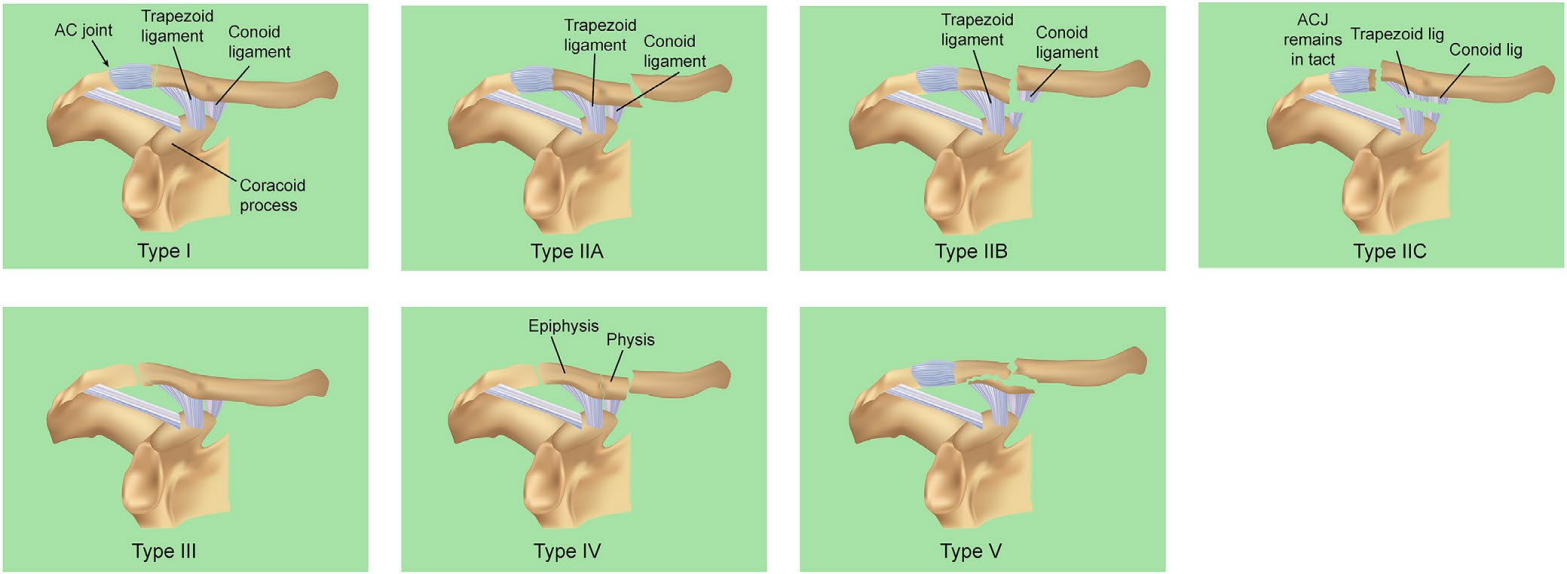
An updated and modified illustration of the Neer classification for distal clavicle fractures. Type I fractures occur distal to the acromioclavicular ligament (i.e., trapezoidal, conical) with minimal displacement, and the acromioclavicular joint remains intact. Type IIA fractures are located medial to the conoid ligament. Type IIB fractures occur between the coracoclavicular ligaments and involve rupture of the conoid ligament, whereas the trapezoid ligament remains intact. Type IIC fractures are lateral to the coracoclavicular ligament, with the acromioclavicular joint remaining intact, and exhibit fracture displacement combined with tears of both the conoid and trapezoid ligaments. Type III fractures extend distal to the coracoclavicular ligament and into the acromioclavicular joint. Type IV fractures are specific to pediatric patients, where the growth plate and scab remain near the acromioclavicular joint, but displacement occurs at the junction of the epiphysis and growth plate. In Type V fractures, a small fragment of the inferior clavicle remains attached to the coracoclavicular ligament.

The Neer classification system [5] remains the primary clinical framework for distal clavicle fractures, originally defining three types based on the coracoclavicular (CC) ligament relationship. Craig [7] later extended it with type IV (pediatric periosteal cuff rupture) and type V (inferior cortical fragment attached to the CC ligament). Levy et al. [8] further subdivided it by introducing type IIC (CC ligament‐distal fracture with intact acromioclavicular joint and conoid/trapezoid ligament tears) (Figure 1). For Neer types I and III fractures without displacement, conservative treatment is recommended. In contrast, surgical intervention is advised for displaced Neer types II (A, B, C), IV (pediatric periosteal cuff rupture with instability), and V (inferior cortical fragment with CC ligament detachment). Treatment options include distal clavicle anatomic splints with or without anchors, clavicle hook plates, clavicle pin wire tension bands, endo‐button fixation, and other internal fixation methods, with the choice of modality primarily based on fracture type and fragment size. In recent years, three‐dimensional CT reconstruction technology has gained popularity for fracture diagnosis and typing due to its superior accuracy and intuitiveness compared with X‐ray diagnosis.

Fracture mapping, also known as a fracture line distribution map, was first introduced by Armitage et al. [9] in 2009. These maps utilize computer software for 3D reconstruction to overlay the fracture lines from multiple cases onto a standardized skeletal model. This approach enables the analysis of fracture line distribution patterns through the application of heatmaps or frequency maps, which illustrate the origin, course, distribution, and comminution of the fracture lines. Consequently, this depiction offers a novel methodology for fracture diagnosis, classification, treatment planning, surgical internal fixation design, identification of preferred fracture sites, and the development of standardized fracture models. However, despite the clinical utility of this technique, its application to distal clavicle fractures remains limited. Current classifications primarily focus on ligament integrity and fragment displacement but lack quantitative characterization of fracture line trajectories.

The objectives of this study were to: (i) characterize the morphological features of distal clavicle fracture lines by examining the displacement patterns associated with these fractures; (ii) identify the location and prevalence of fracture lines in distal clavicle fractures; and (iii) map the distribution of distal clavicle fractures, delineate the areas where fracture lines occur, and refine the classification of bone loss areas using Neer typing based on an analysis of fracture line area distribution.

## 2 Materials and Methods

### 2.1 Patients

This study was approved by the institutional review committee of our hospital (LW20220048). We conducted a retrospective analysis of CT imaging data from patients diagnosed with distal clavicle fractures at Wuxi Ninth People's Hospital between 2019 and 2022. Inclusion criteria are as follows: (i) Radiologically confirmed acute distal clavicle fractures (injury‐to‐diagnosis interval < 3 weeks) with: (a) fracture line extending medial to the coracoclavicular ligaments, (b) involvement of the distal clavicular articular surface or metaphyseal zone; (ii) Preoperative axial CT scans obtained at our institution with slice thickness ≤ 3 mm; (iii) Complete medical records including demographic data and injury mechanism; (iv) Age ≥ 18 years at the time of injury. Exclusion criteria are as follows: (i) Isolated acromioclavicular joint dislocation without fracture; (ii) Pathological fractures (e.g., neoplastic, infectious); (iii) Concomitant ipsilateral scapular/upper limb fractures; (iv) Open fractures or prior surgical intervention on the affected shoulder; (v) CT image artifacts or incomplete scanning sequences; (vi) Preexisting glenohumeral arthritis or systemic rheumatic diseases.

Three senior orthopedic surgeons independently classified fractures using the updated Neer classification [8]. Cases with diagnostic discrepancies underwent adjudication by a panel of two trauma specialists. Of the initial 115 identified cases, 34 were excluded (22 due to suboptimal CT protocols, 12 lost to follow‐up), resulting in a final cohort of 81 patients with 81 fractures.

### 2.2 Image Analysis

Raw CT data were obtained in the axial plane, and DICOM‐formatted CT images of all distal clavicle fractures were imported into FirePlus 3D Medical Design software (Version 2.01, Blackflame Medical Technology Co. Ltd. Shanghai, China) to generate project files. The fracture lines were subsequently analyzed in the axial, sagittal, and coronal planes concurrently to reconstruct 3D models. These digitally reconstructed fracture lines underwent rotation and normalization to align with the 3D clavicle template. Anatomical landmarks, including the bony contour of the entire clavicle and the distal pole, were referenced during the pairing process. The template surfaces were demarcated and subdivided into multiple small, independent surfaces with edges that coincided with the fracture lines of the clavicle fragments. Strokes on the surface were employed to illustrate fracture lines for fragments ≥ 1 cm [3], whereas the surface fill color depicted comminuted zones comprising fragments < 1 cm [3]. All fracture lines and comminuted zones were overlaid onto the template 3D model to create a spatial fracture map. FirePlus 3D Medical Design software was utilized to generate a heatmap based on the spatial frequency of the fracture lines.

### 2.3 Fracture Mapping

**FIGURE 2:**
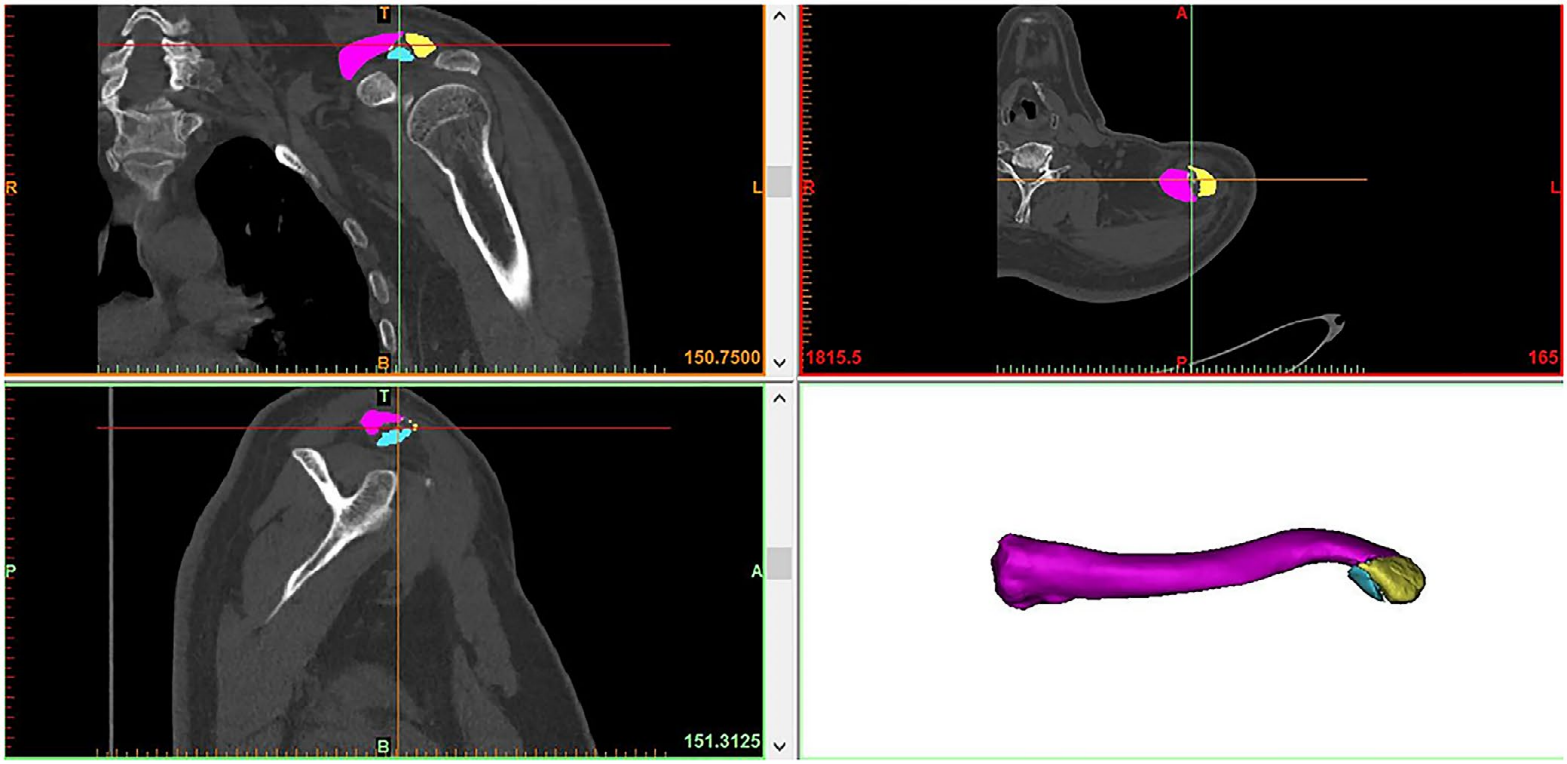
Fracture reconstruction map. The CT imaging data are imported into FirePlus 3D Medical Design software, and the fracture model is reconstructed by aligning specific landmarks: Conus nodus, acromial joint surface, acromial end, and sternal end with appropriate rotation and identification of fracture fragments.

**FIGURE 3:**
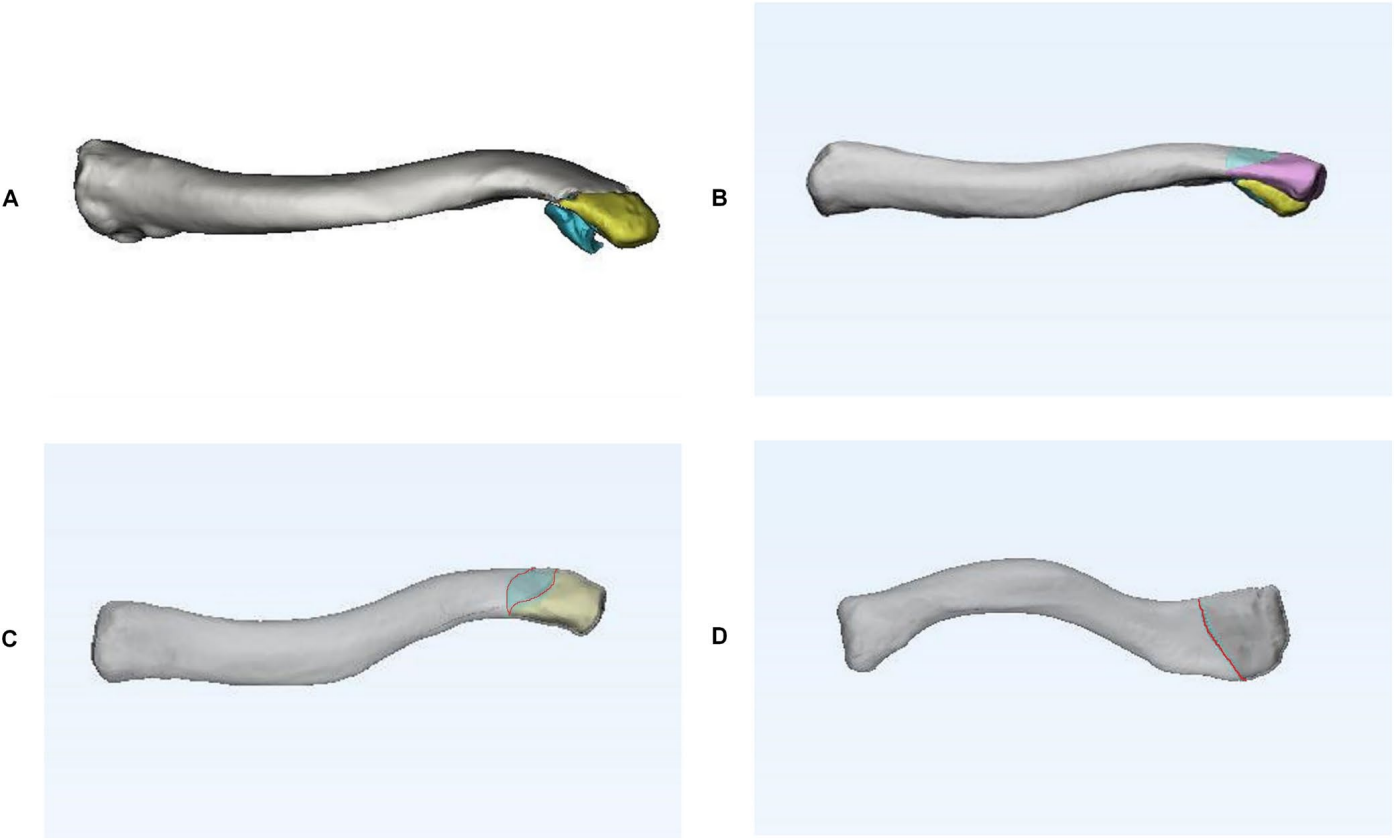
Schematic diagram of the 3D CT imaging simulated repositioning process for distal clavicle fractures. In the case of distal clavicle fracture, each fragment was reconstructed (A) to align with the distal clavicle 3D model (B), followed by virtual repositioning (C). The comminuted regions are delineated with yellow and green outlines (C), whereas the fracture line is indicated by a red line (C, D).

In this study, we employed the fracture mapping method described by Armitage et al. [9]. Briefly, reformatted axial and sagittal images of all the fractures were imported into Adobe Illustrator software and overlaid onto a standard template. This facilitated the editing of fracture lines and crush zones on separate templates for the distal axial and sagittal positions. A crush zone was defined as an area with a crush extent of less than 1 cm^2^. Specific landmarks, including the conical tuberosity, acromion articular surface, acromion end, and bony contour of the sternal end, were aligned to ensure appropriate rotation and identification of fracture fragments (Figure 2). Subsequently, the fracture line and comminuted area were replicated onto the corresponding distal clavicle template. The superposition of all layers resulted in a two‐dimensional map depicting fracture frequency and comminution zone density. To generate the 3D frequency maps, the distal clavicle fracture fragments were reconstructed and virtually restored via FirePlus 3D Medical Design software. The data were then exported to the software, where the reconstructed fragments were rotated, normalized, and horizontally flipped if necessary to achieve the best fit with the 3D model of the distal clavicle (by J.Q.L.). Smooth curves were drawn directly on the model surface to depict fracture lines, and the comminuted zone was marked in each case (Figure 3). By analyzing the overlap of all fracture lines and comminution zones, a 3D map of the distal clavicle fracture line was produced.

**FIGURE 4:**
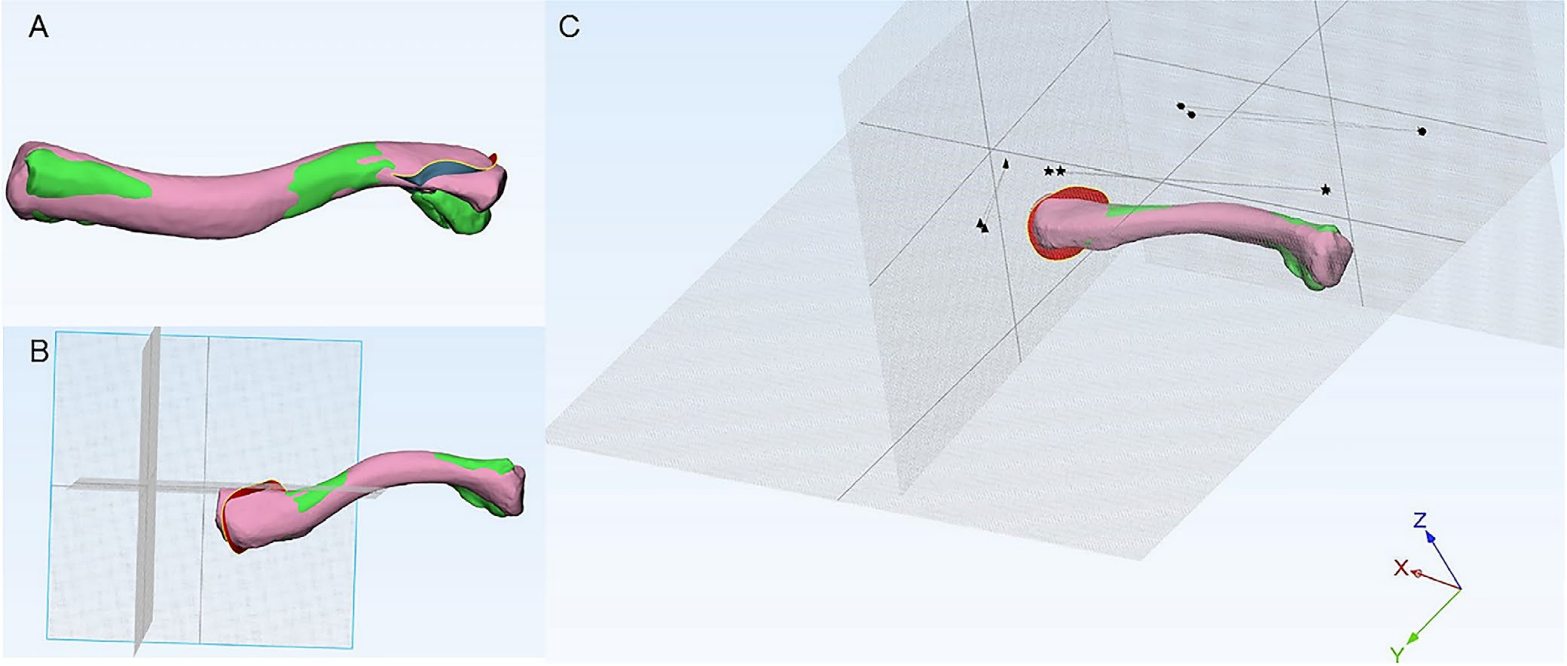
Schematic diagram depicting the process of measuring spatial displacement data for distal clavicle fractures. (A) The 3D fracture simulation imaging map is imported into the standard clavicle 3D imaging map to facilitate virtual reduction. (B) The coronal, sagittal, and horizontal planes are established, with the most distal point of the fracture after reduction serving as the origin. The z‐axis runs parallel to the coronal plane of the body and is positive (+) in the upward direction; the y‐axis runs parallel to the sagittal plane and is positive (+) in the forward direction; and the x‐axis runs parallel to the horizontal plane and is positive (+) in the outward direction. (C) The angle and distance between two specific points, projected onto the planes corresponding to the x‐, y‐, and z‐axes at the distal end of the fracture fragment and at a point two units after repositioning, are measured at the apex of the midpoint angle of the sternal end of the clavicle. The triangular marker in the figure represents the projection point of these three points on the sagittal plane, used to measure anterior–posterior displacement of the fracture. The circular marker denotes the projection point of these three points on the coronal plane, used to measure upward and downward displacement. The pentagram marker indicates the projection point of these three points on the horizontal plane, used to measure shortening and separation of the fracture.

To perform the computer simulation of anatomical reduction, we utilized the software's movement tool and the positioning of two red dots to reposition the three‐dimensional medial fragment model. Following a definitive reduction carried out by one of the authors (J.Q.L.), the process was verified multiple times by an experienced surgeon (M.Z.). Leveraging the 3D rendering program's capability for free 360° rotation in any plane, we analyzed the displacement pattern. We designated the midpoint of the clavicular sternum as a reference point and measured the displacement angle difference between the distal end of the fracture fragment and the corresponding point post‐reduction. Subsequently, with the post‐reduction point as the origin, we established a coordinate system: the z‐axis parallel to the coronal plane of the human body (positive direction upward), the y‐axis parallel to the sagittal plane (positive direction forward), and the x‐axis parallel to the horizontal plane (positive direction outward). We then measured the angle and distance between the maximum displacement point of the fracture block and the corresponding point after reduction, specifically the angle and distance between the projections of these two points on the planes corresponding to the x‐, y‐, and z‐axes. This allowed us to assess the 3D position of the fracture relative to the x‐, y‐, and z‐axes (Figure 4).

### 2.4 Outcome Measures

Primary outcomes focused on the spatial characterization of fracture morphology: (i) fracture line distribution: density heatmaps generated through fracture line superposition across 5 anatomical views (top, bottom, anterior, posterior, lateral); (ii) displacement parameters (measured in mm): (a) vertical displacement (superior/inferior direction), (b) sagittal displacement (anterior/posterior direction), (c) horizontal displacement (medial/lateral direction).

Secondary outcomes included functional anatomy parameters: (i) axial deformity metrics: (a) shortening/separation distance between fracture fragments, (b) angulation degree in three anatomical planes (coronal/sagittal/axial); (ii) biomechanical stability indicators: (a) post‐reduction displacement vectors (x/y/z‐axis), (b) maximum displacement point coordinate deviation.

All quantitative measurements were obtained through FirePlus 3D Medical Design software using its intrinsic coordinate system aligned with anatomical landmarks (conical tuberosity, acromion articular surface). Heatmap densities were normalized to the total fracture line length per unit area.

### 2.5 Statistical Analysis

Patient characteristics and fracture measurements are presented as the means with standard deviations or as proportions. The distribution of fracture lines was analyzed using GraphPad Prism 6.0, applying the chi‐square test or Fisher's exact test to categorical data. The morphologic fracture map analysis and characterization were descriptive in nature.

## 3 Results

**FIGURE 5:**
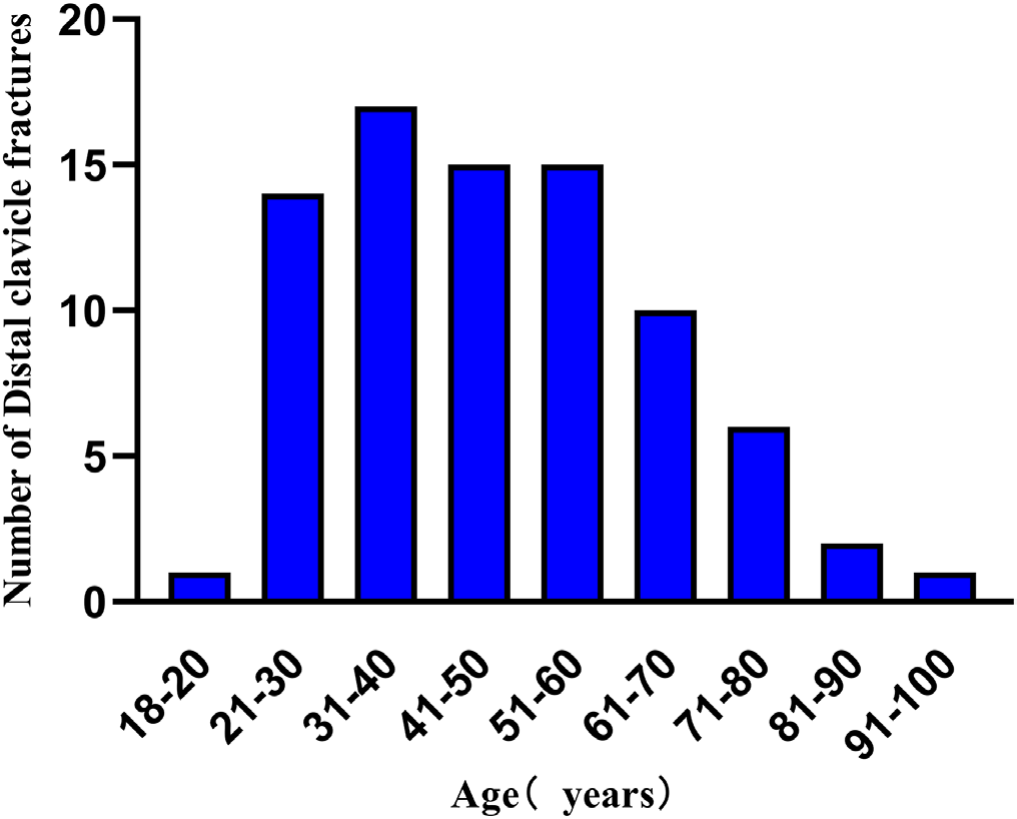
Distribution of fractures by patient age.

Among the 81 patients, 70 (86.4%) sustained fractures between the ages of 21 and 70 years, with the highest incidence observed in patients aged 31 to 40 years (Figure 5). A slightly higher proportion of distal clavicle fractures occurred on the left side compared to the right, and the mean age of patients with left‐sided fractures was greater than that of those with right‐sided fractures. Males predominated in distal clavicle fractures, accounting for 67.9% versus 32.1% in females. Among male patients, the proportion of distal clavicle fractures was equal between the right and left sides, whereas among female patients, the proportion was higher on the left side. According to the updated and modified Neer classification, the distribution of fracture types was as follows: 8 cases (9.88%) of Neer type I, 5 (6.17%) of Neer type IIA, 31 (38.27%) of Neer type IIB, 11 (13.58%) of Neer type IIC, 14 (17.28%) of Neer III, and 12 (14.81%) of Neer V. Neer type IIB was the most prevalent, accounting for 38.27% of all fractures. The mechanisms of injury were as follows: 28 cases (34.57%) due to high falls, 43 cases (53.09%) due to traffic accidents, and 10 cases (12.34%) due to heavy object impacts (Table 1). Three investigators independently reviewed the case records and imaging data to determine the fracture type in each case.

**TABLE 1:** Patient demographics.

Fractures (no.)	81
Age^a^ (years)	47.5 ± 16.6 (18–91)
Sex (no. [%])	
Male	55 (67.9%)
Female	26 (32.1%)
Side of injury (no. [%])	
Left	43 (53.1%)
Right	38 (46.9%)
Fracture type (no. [%])	
Neer I	8 (9.88%)
Neer II	A: 5 (6.17%)
	B: 31 (38.27%)
	C: 11 (13.58%)
Neer III	14 (17.28%)
Neer V	12 (14.81%)
Fracture mechanism (no. [%])	
Fall	28 (34.57%)
Traffic accident	43 (53.09%)
Heavy object injury	10 (12.34%)

^a^ The values are given as the mean and the standard deviation, with the range in parentheses.

### 3.1 Displacement

We observed that 61 patients (75.31%) experienced upper displacement of the fracture fragment, whereas 20 patients (24.69%) experienced lower displacement. The mean distal displacement was 5.10 mm (0.17–15.29 mm) upward and 5.08 mm (0.61–12.34 mm) downward. Additionally, 75 patients (92.59%) exhibited posterior displacement, and 6 patients (7.41%) had anterior displacement of the fracture fragment. The mean distal displacement for posterior and anterior displacement was 6.30 mm (0.24–16.34 mm) and 2.59 mm (0.11–5.64 mm), respectively.

### 3.2 Distraction/Shortening and Angulation

We observed that 74 patients (91.36%) exhibited shortening of the fracture site, whereas 7 patients (8.64%) demonstrated separation. The distal end of the fracture block was shortened by an average of 3.93 mm (0.13–11.56 mm) and separated by an average of 0.48 mm (0.1–0.91 mm). Posterior angulation of the fracture site was observed in 75 patients (92.59%), whereas anterior angulation was noted in 6 patients (7.41%). The mean anterior angulation was 3.79° (0.29°–8.53°), and the mean posterior angulation was 9.24° (0.14°–32.89°). Additionally, 61 patients (75.31%) had superior angulation, and 20 patients (24.69%) had inferior angulation of the fracture site, with mean angles of 6.49° (0.25°–20.47°) and 6.60° (0.76°–24.62°), respectively. The mean angle projected in the plane of the x‐axis was 2.80° (0.07°–7.45°) (Table 2).

**TABLE 2:** Data for 3‐dimensional analysis of displacement patterns for distal clavicle fractures.

Displacement	Number of patients	Distance (mm)	Angulation, degree
		Mean ± SD (range)	
Move forward	6 (7.41%)	2.59 ± 2.15 (0.11–5.64)	3.79 ± 3.04 (0.29–8.53)
Move backward	75 (92.59%)	6.30 ± 3.80 (0.24–16.34)	9.24 ± 6.94 (0.14–32.89)
Move up	61 (75.31%)	5.10 ± 3.26 (0.17–15.29)	6.49 ± 4.74 (0.25–20.47)
Move down	20 (24.69%)	5.08 ± 3.72 (0.61–12.34)	6.60 ± 6.22 (0.76–24.62)
Shortening	74 (91.36%)	3.93 ± 2.38 (0.13–11.56)	
Distraction	7 (8.64%)	0.48 ± 0.39 (0.1–0.91)	

### 3.3 Fracture Mapping

**FIGURE 6:**
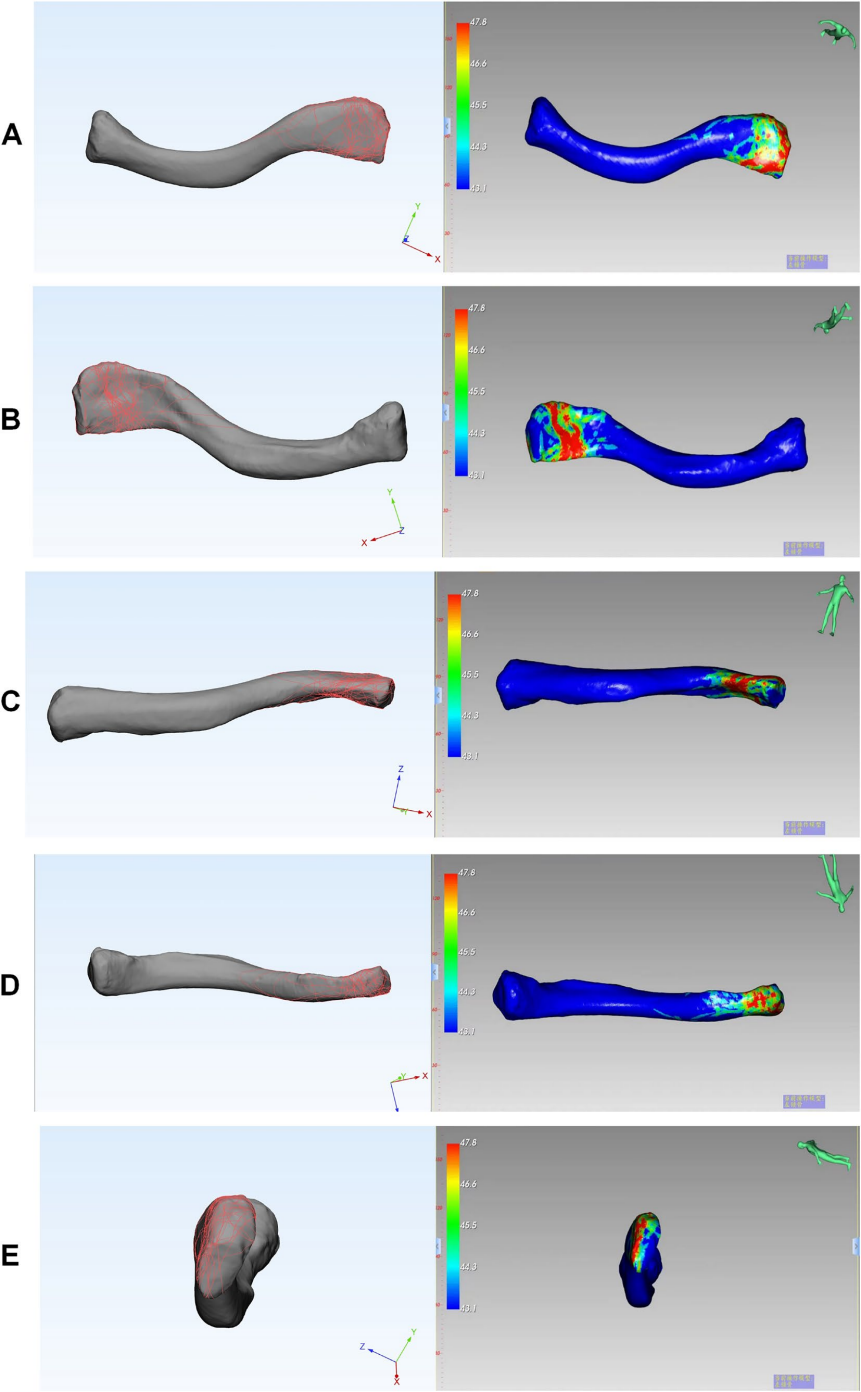
Fracture and heatmaps illustrating the location, distribution, and frequency of all 81 distal clavicle fractures. (A)Fracture map (left) and heatmap (right) of the top view (TV). (B) Fracture map (left) and heatmap (right) of the bottom view (BV). (C) Fracture map (left) and heatmap (right) of the anterior view (AV). (D) Fracture map (left) and heatmap (right) of the posterior view (PV). (E) Fracture map (left) and heatmap (right) of the lateral view (LV). The red color represents a higher frequency of fracture line density.

In the top view, the majority of distal clavicle fracture lines were located far from the distal conical tuberosity, with the highest density observed at the acromion and the posterior distal end of the clavicle, particularly near the acromioclavicular joint surface. Here, most fracture trajectories were transverse lines perpendicular to the horizontal axis of the clavicle, with a narrow span. Conversely, fracture lines were less prevalent medially to the distal conical tuberosity and exhibited an oblique orientation, extending from the medial‐superior to the lateral‐inferior side with a wider span. A few fracture line trajectories extended along the anterior edge of the distal clavicle toward the acromioclavicular joint. The distal clavicle fracture heatmap corroborated these findings, showing a dense area of fracture lines and their extension direction from medial to the acromioclavicular joint, resembling the mathematical symbol for greater than or equal to (≥) (Figure 6A). In the bottom view, the fracture line concentration area was nearly perpendicular to the diagonal line, and the heatmap exhibited a pattern similar to a 70° clockwise rotation of the approximate sign (≈) (Figure 6B). In the anterior view, fracture lines were primarily concentrated on the inner one‐third of the deltoid origin, with the dense area on the heatmap located accordingly (Figure 6C). In the posterior view, fracture lines were mainly concentrated at the insertion of the trapezius muscle, with the heatmap indicating a dense area on the outer one‐third of this insertion (Figure 6D). Finally, in the lateral view, fracture lines were primarily concentrated in the upper one‐third of the distal clavicle, with the heatmap demonstrating a dense area in the upper one‐quarter of the clavicle (Figure 6E).

### 3.4 Case Presentation

**FIGURE 7:**
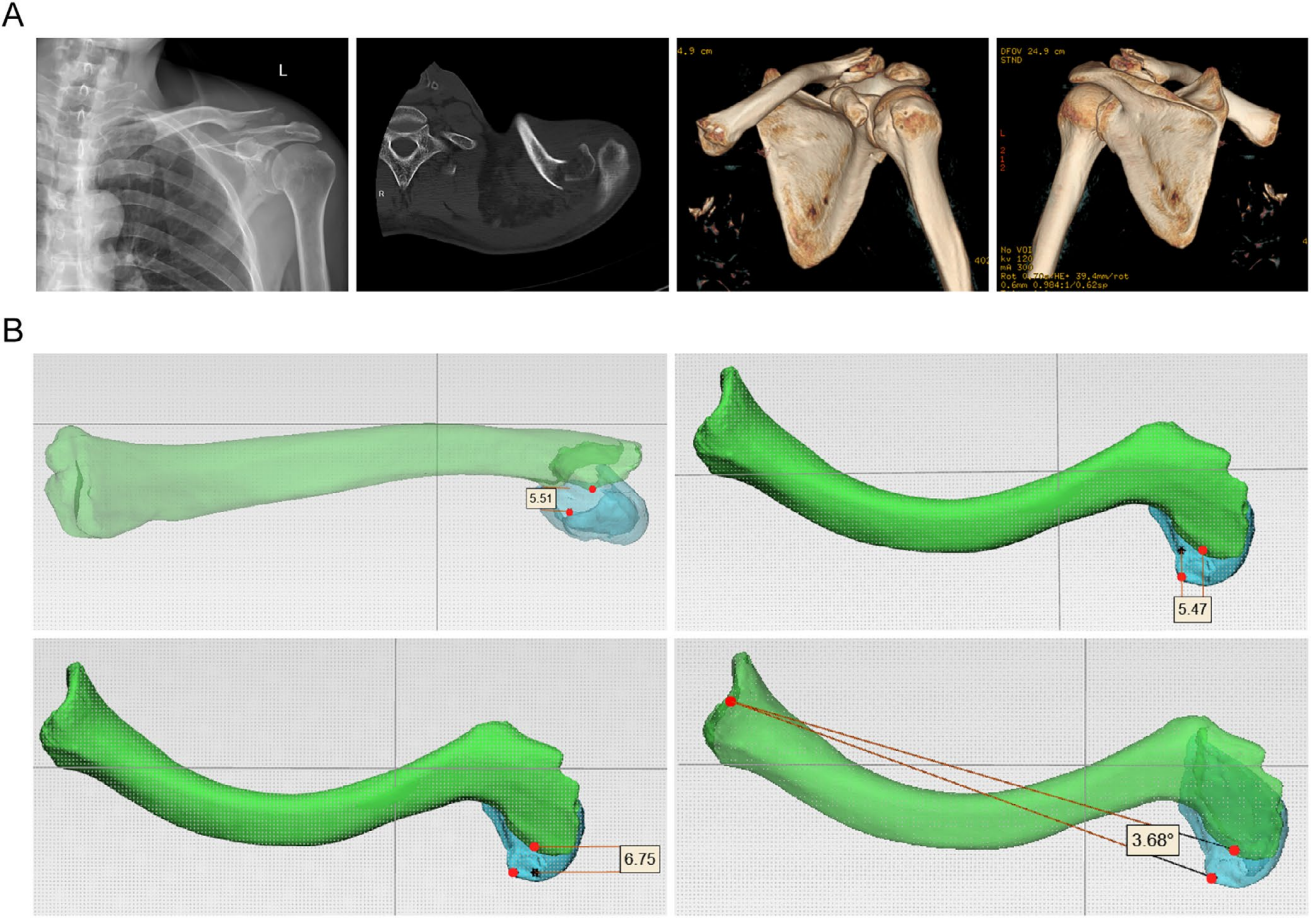
A 58‐year‐old male with a Neer IIB distal clavicle fracture of the left shoulder. (A) Plain radiograph and CT scan images, (B) simulated images using 3D rendering software.

Case 1 A 58‐year‐old male sustained a left distal clavicle fracture due to a fall. X‐rays revealed a mildly displaced fracture, while CT scans showed posterior displacement of the medial fragment, consistent with a Neer IIB fracture (Figure 7A). Measurements indicated that the medial fragment was displaced 5.51 mm superiorly and 6.75 mm posteriorly, with a shortening of 5.47 mm at the fracture site and a posterior angulation of 3.68° (Figure 7B).

**FIGURE 8:**
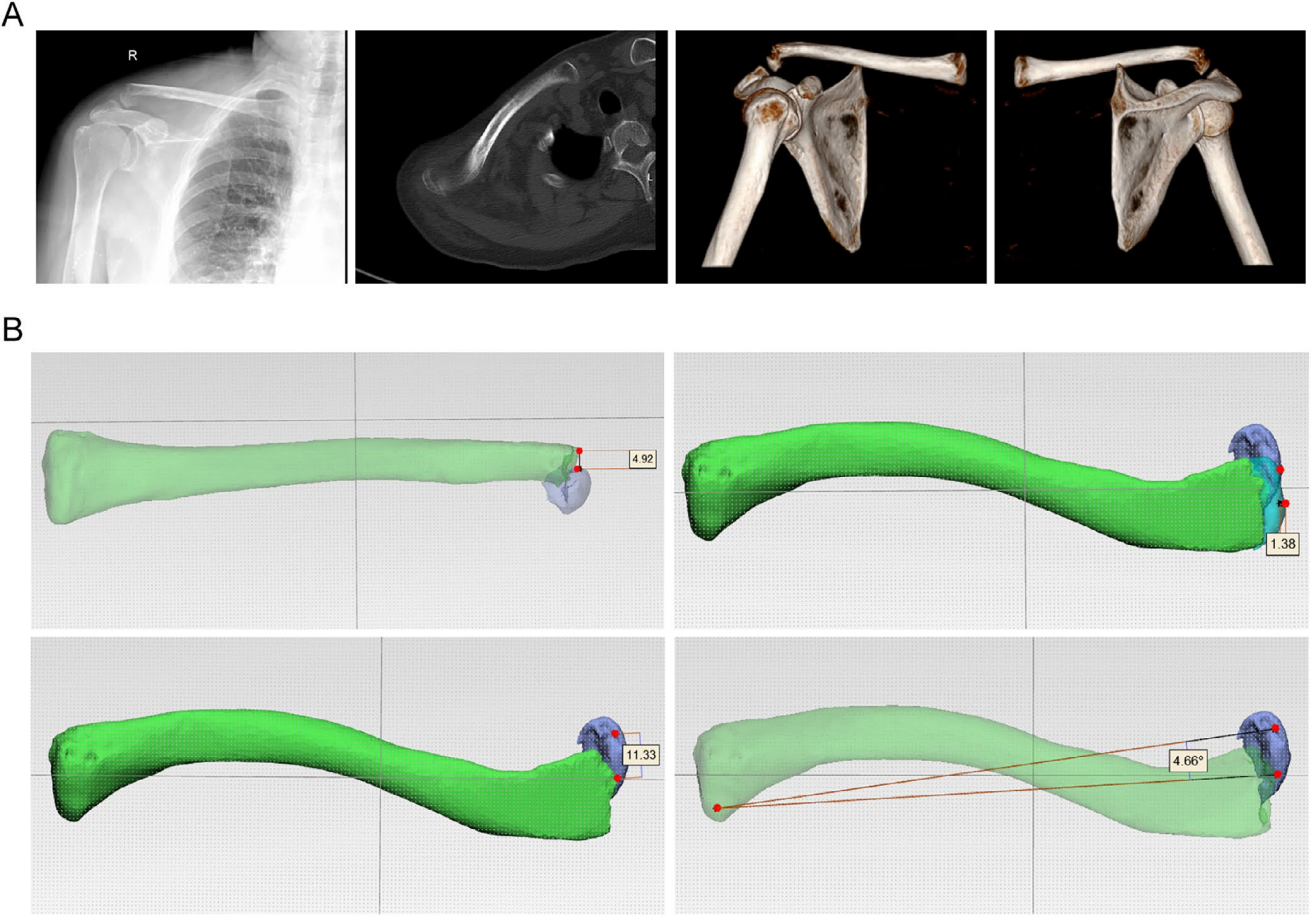
A 63‐year‐old female with a Neer IIC distal clavicle fracture of the right shoulder. (A) Plain radiograph and CT scan images; (B) simulated images generated with 3D rendering software.

Case 2 A 63‐year‐old female presented with a right distal clavicle fracture following a fall. X‐rays demonstrated superior displacement of the medial fragment, and CT scans showed superior and posterior displacement, with the fracture line located distal to the coracoclavicular ligament, confirming a Neer IIC fracture (Figure 8A). Measurements showed that the medial fragment was displaced 4.92 mm superiorly and 11.33 mm posteriorly, with a shortening of 1.38 mm at the fracture site and a posterior angulation of 4.66° (Figure 8B).

**FIGURE 9:**
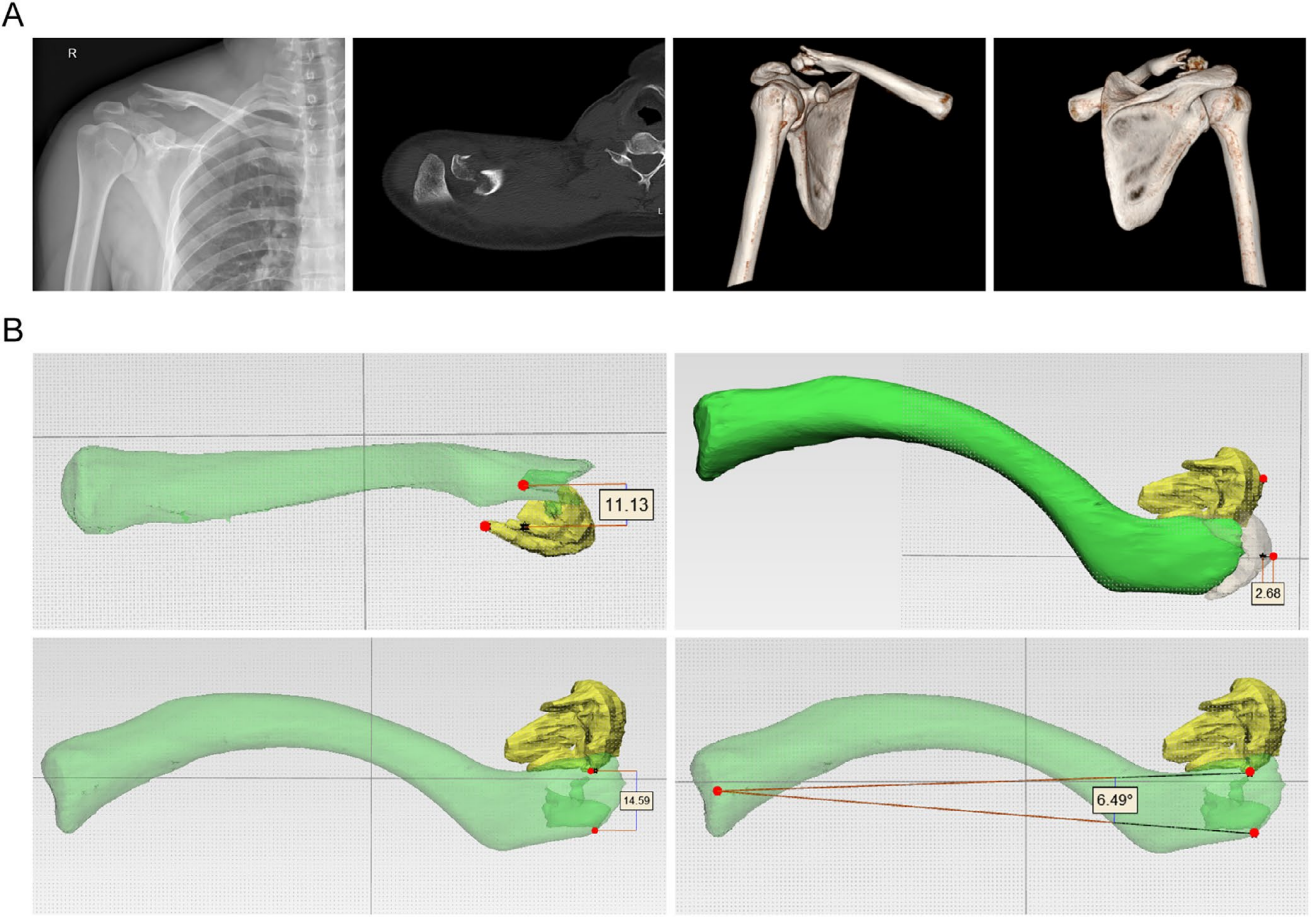
A 28‐year‐old male with a Neer V distal clavicle fracture of the right shoulder. (A) Plain radiograph and CT scan images, (B) simulated images created using 3D rendering software.

Case 3 A 28‐year‐old male suffered a right distal clavicle fracture in a motor vehicle accident. X‐rays revealed a comminuted distal clavicle fracture with superior displacement of the medial fragment, and the coracoclavicular ligament was attached to the inferior fragment. CT scans confirmed superior and posterior displacement of the medial fragment, consistent with a Neer V fracture (Figure 9A). Measurements indicated that the medial fragment was displaced 11.13 mm superiorly and 14.59 mm posteriorly, with a shortening of 2.68 mm at the fracture site and a posterior angulation of 6.49° (Figure 9B).

## 4 Discussion

Distal clavicle fractures, accounting for 10%–30% of all clavicle fractures, pose management challenges due to the difficulty in distinguishing subtle fracture type variations that may indicate instability. While stable fractures typically heal with non‐surgical treatment, unstable fractures require longer healing times and have higher nonunion rates. Despite this, no comprehensive mapping study has examined all types of distal clavicle fractures, and current classifications are limited in guiding treatment decisions. This study presents a fracture map analysis via CT imaging, revealing that most displaced distal clavicle fractures exhibit multidirectional displacements and angulations. Our analysis of 81 cases identified Neer IIB as the predominant type, with fracture lines concentrated in the distal one‐third of the distal clavicle, extending from superior to lateral inferior. These findings underscore the importance of considering multidirectional displacement and angulation in treatment planning.

### 4.1 Injury Mechanisms and Classification of Distal Clavicle Fractures

The mechanism of injury for distal clavicle fractures typically involves a medially directed force resulting from a fall or traumatic event, where the lateral shoulder (tip of the acromion) impacts the ground or a rigid surface [10, 11]. Specifically, when the arm is in an adducted position, this force is transmitted through the acromioclavicular (AC) joint to the distal clavicle and coracoclavicular (CC) ligaments, owing to the stability of the sternoclavicular joint [11]. The bone fails under tension superiorly and compression inferiorly, leading to various fracture types, ranging from simple transverse fractures to comminuted fractures with multiple fragments. Stable distal clavicle fractures generally heal successfully with nonoperative treatment and yield satisfactory clinical results. Conversely, unstable distal clavicle fractures have historically posed challenges, as they are often associated with prolonged healing times and non‐healing rates of 18%–43% without surgical intervention, necessitating surgical management. However, the diagnosis and treatment of these fractures are often challenging due to the difficulty in identifying fracture patterns indicative of instability [1]. Understanding these injury patterns and their imaging characteristics is crucial for appropriate decision‐making and management of distal clavicle fractures [1, 3, 12, 13].

Traditionally, the Neer classification system and its modified version proposed by Craig have been used to assess fracture stability, focusing solely on coronal plane assessment. Robinson [8] introduced a classification for all clavicle fractures based on the displacement of major fragments (> 100% or < 100% translation), categorizing them into subgroups A and B but did not specify the direction of displacement. Bishop et al. [14] emphasized that fracture stability, rather than the Neer classification or the size of the distal fragment, is a key factor in treatment decisions for distal clavicle fractures. Assessing displacement patterns using only the coronal plane may overlook the horizontal displacement of the fracture fragment. While previous studies have primarily focused on vertical displacement in the coronal plane, horizontal displacement in the sagittal plane has been underappreciated. Determining fracture patterns and stability to guide treatment decisions remains challenging [1]. Based on these observations, Cho et al. [15] analyzed 37 patients with distal clavicle fractures and evaluated horizontal displacement of the fracture block, reporting that most medial blocks are angled backward. However, distal clavicle fractures occur in three‐dimensional space, and the fracture should be assessed in terms of the overall distance and angle of displacement of the fracture block in all planes. The multidirectional displacement and angulation observed in our study suggest that the fracture pattern is influenced by both shear and compression forces. This understanding is vital for assessing fracture stability and guiding appropriate treatment modalities.

### 4.2 Fracture Mapping Analysis of Distal Clavicle Fractures

Fracture mapping, also known as a fracture line distribution map, was first introduced by Armitage et al. [9] in 2009. This technique utilizes computer software for three‐dimensional reconstruction, overlaying fracture lines from multiple cases onto a standard bone model. By analyzing the distribution of these lines using heatmaps or frequency diagrams, fracture mapping depicts the origin, course, distribution, and comminution of fractures, aiding orthopedic surgeons in a more three‐dimensional analysis of fracture characteristics. Current research on fracture mapping encompasses intra‐articular distal radius fractures [16], tibial plateau fractures [17], pilon fractures [18, 19], proximal humerus fractures [20], scapular fractures [21], patella fractures [22], and Hoffa fractures [23]. Van et al. [24] employed 3D CT to evaluate the fracture pattern of clavicle fractures. However, no studies on fracture mapping of distal clavicle fractures have been conducted to date. We applied the fracture mapping technique described by Armitage et al. [9] to 81 isolated distal clavicle fractures, representing, to our knowledge, the largest series reported in this field. This approach aimed to enhance our understanding of this fracture injury. We developed a quantitative evaluation method that combines uniquely derived fracture line features with a qualitative assessment of fracture patterns, including two‐ and three‐dimensional analyses, to provide a comprehensive description of distal clavicle fracture morphology. Fracture frequency and heatmaps indicate that most distal clavicle fractures occur distal to the conical tuberosity, particularly at the acromion end, extending from superior to lateral inferior.

Using 3D CT followed by post‐fracture reduction clavicle reconstruction, we observed that 61 (75.31%) patients had upward displacement and angulation of fracture fragments, while 20 (24.69%) exhibited downward displacement and angulation. Additionally, 75 patients (92.59%) showed posterior displacement and angulation of the fracture fragments. Notably, 74 (91.36%) patients exhibited shortening at the fracture site, and distal clavicle fractures displayed displacement and angulation in multiple orientations. Therefore, attention to the fractures in all orientations is crucial during repositioning.

Our 3D analyses have identified two key biomechanical factors that predict therapeutic failure in distal clavicle fractures: posterior displacement and posterior angulation, which were observed in 92.59% of cases. This particular spatial configuration exerts detrimental shear forces on the coracoclavicular ligament complex, creating a pathomechanical environment that corresponds to the 31% nonunion rate reported in conservatively managed Neer type IIB fractures [25]. The study by Cho et al. [15] similarly demonstrates that 3D evaluation of displacement patterns in distal clavicle fractures is valuable for assessing fracture stability and informing treatment decisions. Understanding the distal clavicle fracture situation (simple/comminuted), fracture line alignment, and fracture displacement pattern in high‐incidence areas will assist orthopedic surgeons in analyzing fracture characteristics more three‐dimensionally. This, in turn, will facilitate the selection of an appropriate treatment plan, optimal surgical internal fixation design, and improved patient outcomes.

As demonstrated in this study, when an unstable fracture is suspected based on plain radiographs, a more accurate assessment of the severity and orientation of the distal clavicle fracture should be made using CT. CT can assess horizontal instability and detect underestimated or misdiagnosed distal clavicle fractures. In cases of uncertain horizontal displacement, CT may be considered for the most accurate assessment of clavicle displacement patterns. Furthermore, CT can aid in surgical planning and determining the adequacy of crushed bone at the time of surgical fixation.

Additionally, the distal clavicle fracture map developed in this study has contributed to a more comprehensive and descriptive classification system. We found that the fracture line orientation was both continuous and multiplanar. The fracture types identified in our study may facilitate the development of fixation concepts related to distal clavicle fractures. With a more accurate understanding of distal clavicle fracture morphology, further biomechanical studies can be conducted to better model clinical injury patterns. To our knowledge, this is the first time that two‐ and three‐dimensional fracture speciation techniques, combined with CT tomographic reconstruction, have been used to elucidate common fracture types of isolated distal clavicle fractures.

Three‐dimensional fracture labeling has been extensively used to improve the understanding of various skeletal injury patterns [16, 17, 18, 19, 20, 21, 22, 23]. Although time‐consuming and technically demanding, 3D markings are more accurate in depicting fracture patterns than 2D markings and provide essential additional information for preoperative planning. In this study, 3D distal clavicle fracture maps showing the surface anatomy of common fracture lines and joint comminution allowed for the design of the best surgical approach, maximizing fracture visualization and minimizing the risk of injury to nearby critical structures. While the sagittal 2D map reflects the depth of joint comminution, the 3D fracture map shows the anatomical location and surface area. Thus, the 2D map reveals the “inside” of the fracture, and the 3D map shows the “outside”; combining these techniques enhances the completeness and accuracy of our understanding of distal clavicle fractures.

### 4.3 Prospect of Clinical Application

The fracture mapping technique and 3D analysis presented in this study hold significant potential to enhance clinical decision‐making and surgical precision in managing distal clavicle fractures. By defining the spatial relationship between fracture lines, displacement patterns, and anatomical landmarks, this approach improves preoperative recognition of instability and guides individualized treatment strategies. The ability to visualize multidirectional displacement and angulation through 3D CT reconstruction enables surgeons to distinguish stable from unstable fractures more reliably, particularly in cases where conventional radiographs underestimate fracture complexity. For example, identifying posterior displacement or angulation on preoperative imaging may prompt earlier surgical intervention for Neer IIB fractures, potentially reducing nonunion rates and associated complications. Furthermore, fracture maps serve as a valuable educational tool for trainees, facilitating a deeper understanding of injury mechanics and fracture patterns. From a societal perspective, optimizing fracture classification and treatment algorithms could reduce the economic burden of prolonged immobilization, repeat imaging, and revision surgeries while improving functional outcomes and return‐to‐work timelines for patients. Future integration of these mapping techniques with biomechanical modeling or artificial intelligence tools may further refine implant design and fixation strategies, advancing personalized care for distal clavicle fractures.

### 4.4 Strengths and Limitations

This study elucidates for the surgeon a better understanding of the characteristics inherent to distal clavicle fractures, an understanding deemed essential for optimal preoperative planning, encompassing the selection of surgical access and fixation structures. Our findings significantly contribute to the broader understanding of fracture morphology, thereby informing the design of fracture classification systems and biomechanical studies, ultimately guiding treatment strategies.

However, it is imperative to acknowledge the noteworthy limitations of the current study. Firstly, the sample size remains modest, and both the fracture line frequency map and heatmap are derived from limited sample statistics. Furthermore, the study population consisted solely of inpatients with distal clavicle fractures, excluding those diagnosed in outpatient settings, which introduces a degree of bias into the statistical results. Additionally, in comparison to community hospitals, the patients within our study exhibited a higher prevalence of complex distal clavicle fractures, potentially skewing the findings. Secondly, challenges arose in marking two corresponding points prior to and following the repositioning of fracture models to analyze the displacement patterns of distal clavicle fractures, which are crucial for providing guidance on anatomical repositioning. The manual nature of this marking process raises the possibility of selection bias regarding the two corresponding points. Thirdly, this investigation was a three‐dimensional reconstruction study utilizing CT images; yet it did not encompass a comparative analysis of the fracture line distribution across various types of distal clavicle fractures. Given the anatomical variability of the distal clavicle, certain fracture images did not align precisely with the three‐dimensional distal clavicle model, resulting in potential discrepancies between the fracture lines depicted on the model and the actual fracture patterns. Lastly, the mechanisms of injury associated with distal clavicle fractures and their correlation with the fracture maps have not been thoroughly examined. Future studies could benefit from an increased sample size and a detailed exploration of the distinctions in the distal clavicle fracture map, considering the mechanisms of injury, varying age groups, gender, and fracture patterns.

## 5 Conclusion

In conclusion, this study emphasizes the need for 3D analysis to understand and manage distal clavicle fractures. The application of fracture mapping technology has provided valuable morphological insights that can guide preoperative planning and surgical decision‐making. However, further research is needed to overcome the limitations of this study and to develop more comprehensive classification systems and targeted fixation strategies for distal clavicle fractures. By addressing these challenges, we can improve patient outcomes and advance our understanding of this complex fracture type.

## Author Contributions

**Jinquan Liu, Jingyi Mi** and **Yesheng Jin:** conceptualization and formal analysis. **Jinquan Liu, Jingyi Mi, Yesheng Jin, Fang Lin, Yongwei Wu** and **Yunhong Ma:** data curation. **Jinquan Liu, Jun Liu, Zhonghua Xu, Li Tang, Aiping Zhu**, and **Danfeng Jing:** methodology and 3D reconstruction. **Ming Zhou and Yongjun Rui:** supervision. **Jinquan Liu, Jingyi Mi, Yesheng Jin, Fang Lin**, and **Ming Zhou:** writing – original draft. **Jinquan Liu, Jingyi Mi, Yesheng Jin, Ming Zhou** and **Yongjun Rui:** writing – review and editing. **Yongwei Wu, Zhonghua Xu, Li Tang** and **Jinquan Liu:** statistical methods. All authors contributed to the article and approved the submitted version.

## Ethics Statement

The study was approved by the Ethics Committee of Wuxi Ninth People's Hospital Affiliated to Soochow University (LW20220048).

## Consent

Written informed consent for publication was obtained from all participants.

## Conflicts of Interest

The authors declare no conflicts of interest.

## Supporting information


Data S1.

Data S2.

## Data Availability

The datasets used and/or analyzed during the current study are available from the corresponding author on reasonable request.
