# Supplementary material for: Three‐Dimensional Mapping of Distal Clavicle Fractures: Displacement Patterns and Clinical Implications for Surgical Management
Source: Orthop Surg. 2025 Mar 24;17(6):1656–68. doi: 10.1111/os.70033 (PMC12146135; doi:10.1111/os.70033)
Supplement: Supplementary file 1 — Data S1. [file OS-17-1656-s002.docx]

**Medical Ethics Review Opinion**

**Wuxi Ninth People's Hospital (Wuxi Orthopedic Hospital)**

**Medical Ethics Committee**

**Ethics Review Opinion**

**Number**: LW20220048

**Project Name**:
Analysis of Fracture Lines of Distal Clavicle Fractures

**Project Type**:
Clinical New Technology

**☑ Research**

**Applicant**:
Jingyi Mi

**Department**:
Sports Medicine

**Review Category**:
Initial Application for a New Project

**Review Method**:
Expedited Review

**Review Date**:
January 6, 2023

**Review Committee Members**:

**Review Materials**:
Review Application, Research Protocol, Informed Consent Forms

**Review Opinion**:
Upon review, the experimental design and implementation plan of the project " Analysis of Fracture Lines of Distal Clavicle Fractures" have fully considered principles of safety and fairness. This project is in compliance with the "Ethical Review of Biomedical Research Involving Human Subjects (Trial)" issued by the Ministry of Health, as well as the relevant provisions of the Helsinki Declaration regarding biological human experimentation. The research content does not pose harm or risk to the subjects.

After review by the Ethics Committee, there are no conflicts of interest regarding the research content and results. Therefore, it is approved that the fieldwork of this study can proceed as planned.

**Signature of Chairman/Vice Chairman**:

**Wuxi Ninth People's Hospital Medical Ethics Committee**
