# Supplementary material for: Three‐Dimensional Mapping of Distal Clavicle Fractures: Displacement Patterns and Clinical Implications for Surgical Management
Source: Orthop Surg. 2025 Mar 24;17(6):1656–68. doi: 10.1111/os.70033 (PMC12146135; doi:10.1111/os.70033)
Supplement: Supplementary file 2 — Data S2. [file OS-17-1656-s001.pdf]

科研伦理审查意见

无锡市第九人民医院（无锡市骨科医院）

医学伦理委员会

伦理审查意见

编号：LW20220048

项目名称	锁骨远端骨折的骨折情况分析		
项目类型	☐临床新技术　　☑科研		
申请者	糜菁熠	科室	运动医学科
审查类别	首次申请新项目	审查方式	快速审查
审查时间	2023-01-06		
审查委员			
审查材料	☑审查申请书　☑研究方案　☑ 知情同意书		

审查意见

经审查，锁骨远端骨折的骨折情况分析 项目的实验设计和实施方案充分考虑了安全性和公平性原则。此项目符合卫生部《涉及人的生物医学研究伦理审查办法（试行）》及赫尔辛基宣言关于生物学人体试验的相关规定，研究内容不构成对受试者的伤害和风险。

经伦理委员会审核，研究内容和结果不存在利益冲突。同意该项研究的现场工作按计划进行。

主任委员/副主任委员签字

[illegible]

无锡市第九人民医学伦理委员会

日期：2023 年 1 月 9 日

（印章：无锡市第九人民医院（骨科医院）伦理委员会）

扫描全能王 创建
